# Micro- and Macroabrasion in the Esthetic Zone: A Narrative Review and Case Study

**DOI:** 10.3390/dj13050183

**Published:** 2025-04-23

**Authors:** Jose Villalobos-Tinoco, Carlos A. Jurado, Silvia Rojas-Rueda, Nechama S. Citrin, Staley Colvert, Jose Luis Gutierrez-Quintero, Salwa Mekled

**Affiliations:** 1 Postgraduate Program in Periodontology and Implant Dentistry, School of Dentistry, National University of Rosario, Rosario 3160, Argentina; 2 Independent Researcher and Clinician, Culiacan 80030, Mexico; 3 Division of Operative Dentistry, Department of General Dentistry, College of Dentistry, The University of Tennessee Health Science Center, Memphis, TN 38104, USA; 4 School of Dentistry, Ponce Health Sciences University, Ponce 00716, Puerto Rico; 5 Division of Dental Biomaterials, School of Dentistry, The University of Alabama at Birmingham, Birmingham, AL 35233, USA; 6 Department of General Dentistry, College of Dentistry, The University of Tennessee Health Science Center, Memphis, TN 38104, USA; 7 Residency in Periodontics, Autonomous University of Baja California, Tijuana 22390, Mexico; 8 Department of Restorative Dentistry, Temple University Kornberg School of Dentistry, Philadelphia, PA 19140, USA

**Keywords:** microabrasion, macroabrasion, resin infiltration, esthetic zone

## Abstract

**Background:** Micro- and macroabrasion represent a minimally invasive treatment approach for stained teeth in the esthetic zone. Diagnosing the type of stain is crucial for selecting the appropriate treatment approach. These clinical procedures involve several meticulous steps that may be confusing for less experienced clinicians. **Methods:** The objective of this article is to provide an updated review of the literature on the clinical procedures for micro- and macroabrasion and to present a clinical case in which a minimally invasive macroabrasion procedure was applied to treat a female patient seeking to remove stains from her anterior teeth. Preliminary reviews were conducted of existing case reports and reviews evaluating the clinical procedures and outcomes of micro- and macroabrasion. **Results:** A review of the literature reveals minor differences in how stains on anterior teeth are addressed. Depending on the depth of the stain, microabrasion is typically used for superficial stains, while macroabrasion is employed for deeper stains. Clinicians often combine micro- or macroabrasion with tooth whitening procedures. Literature reviews agree that micro- and macroabrasion techniques are effective minimally invasive approaches that yield high esthetic results. The case study demonstrated each clinical step of microabrasion, achieving results that fully satisfied the patient’s esthetic demands. **Conclusions:** Micro- and macroabrasion can be effective and minimally invasive methods for treating stained anterior teeth. Superficial stains can be treated with microabrasion, while deeper stains may require macroabrasion. In some clinical scenarios, tooth whitening can also be combined with these treatments.

## 1. Introduction

Even minor defects located within the smile zone are often readily noticed by patients and can result in significant esthetic and psychological concerns. Research indicates that enhancements in smile esthetics are closely associated with improvements in self-esteem, emotional well-being, and overall quality of life [1,2]. Addressing such dental imperfections requires a tailored approach based on the size, depth, and type of lesion present. For small, localized defects, conservative options such as direct resin composite restorations are typically sufficient, offering an effective and esthetic solution with minimal intervention. In contrast, larger or structurally compromising defects may necessitate more extensive treatment, such as indirect restorations or full-coverage crowns. Despite the availability of various restorative techniques, modern dental practice emphasizes the importance of minimally invasive dentistry. This philosophy prioritizes the preservation of healthy tooth structure while still aiming to meet both the functional and esthetic expectations of the patient [3,4]. Clinicians are encouraged to evaluate each case comprehensively, considering factors such as occlusion, tooth vitality, and patient-specific esthetic demands. When feasible, the use of minimally invasive treatments not only supports long-term oral health but also aligns with patient preferences for less aggressive and more cost-effective solutions.

Managing stains on anterior teeth can be clinically challenging, as it requires a detailed assessment of their underlying cause prior to initiating treatment [5]. Available treatment options range from basic procedures like scaling and polishing to more conservative approaches such as microabrasion or macroabrasion, as well as restorative solutions including resin composites, composite veneers, ceramic veneers, or even full-coverage crowns [6,7]. The appropriate treatment is determined by the nature of the stain, whether it is external (limited to enamel) or internal (involving the dentin). Additionally, stains can be further classified based on whether their origin is local or systemic, as outlined in Table 1 [8].

Microabrasion and macroabrasion are two types of minimally invasive approaches used to treat stained teeth in the smile zone (Table 2). Depending on the size of the lesion, clinicians can choose either technique. Microabrasion is a technique that removes only the outer surface of the enamel using acids such as a mixture of 18% hydrochloric acid and pumice, or 6.6% to 10% hydrochloric acid with silica carbide particles, or even 37% phosphoric acid gel with extra fine pumice [9,10,11]. Macroabrasion is another conservative treatment option for deeper and more severe tooth stains that cannot be effectively addressed with microabrasion or resin infiltration. However, microabrasion remains a more conservative approach compared to traditional veneers or crown restorations. This is because microabrasion does not involve a defined tooth preparation design. Although diamond burs in a high-speed handpiece are used during the procedure, only light and intermittent pressure is applied to the stained surface to selectively remove the discoloration. It is also important to keep the tooth hydrated throughout the process. After the stain is removed, a direct resin composite is applied to the treated area [12,13].

Both micro- and macroabrasion can be considered conservative treatment approaches, as these techniques remove only the stained areas or defects on the outer surface of the teeth. Tooth whitening can also be combined with these treatments to further reduce staining and enhance the overall appearance of the dentition [14]. However, even when combined, these techniques have limitations and may not be effective for deep stains or developmental defects. In such cases, patients must be clearly informed from the outset that traditional treatments, such as ceramic veneers or crowns, may be the only viable option [15,16].

Direct resin composites in the anterior zone provide high esthetic results [17] and long-term survival rates [18], making them a suitable option for use in conjunction with microabrasion treatment. A recent study evaluated the longevity of direct resin composite restorations. Specifically, a retrospective clinical study assessed anterior composite restorations (Class III, Class IV, and veneers) with a 15-year follow-up. The results showed an annual failure rate of only 2.4% for Class III and IV restorations, and an 85% success rate for direct composite veneers at 5 years [19]. Furthermore, a recent systematic review and meta-analysis of the literature on the success rate of resin composite anterior restorations in localized wear areas reported a 93% survival rate over a 2–7-year period. The authors concluded that anterior composite restorations offer a high success rate [20].

Several clinical steps are required to perform either micro- or macroabrasion in minimally invasive restorative procedures in the esthetic zone, but these steps lack clarity in the literature and may be confusing for young clinicians. Therefore, this manuscript provides a concise review of the micro- and macroabrasive protocols used in case reports and studies evaluating their long-term success. It also includes a case study that outlines the clinical evaluation and steps in the clinical workflow necessary to achieve optimal esthetic results that meet the patient’s functional and esthetic needs.

## 2. Materials and Methods

### 2.1. Review of the Literature

Microabrasion and macroabrasion are minimally invasive dental techniques aimed at preserving as much healthy tooth structure as possible. However, the clinical procedure may be challenging for young clinicians due to the multiple steps involved. To address this, a comprehensive search was conducted in January 2025 using MEDLINE (PubMed), Google Scholar, Semantic Scholar, and ResearchGate to identify relevant articles published between 1980 and January 2025. The search terms used were “microabrasion” OR “macroabrasion”. Case reports, case studies, retrospective studies, and systematic reviews were considered. Only articles written in English were included. The articles were divided into two tables. The first table included only manuscripts with clinical studies, and the eligibility criteria required cases that describe every single clinical step of the procedure. Reports that involve other dental procedures, such as implant therapy or ceramic veneers and crowns, were not included. The second table focused solely on reviews related to the procedure, with eligibility criteria limited to systematic reviews, scoping reviews, and narrative reviews. The study selection process was conducted independently by two reviewers, with any disagreements resolved by a third reviewer.

### 2.2. Case Study

**Figure 1:**
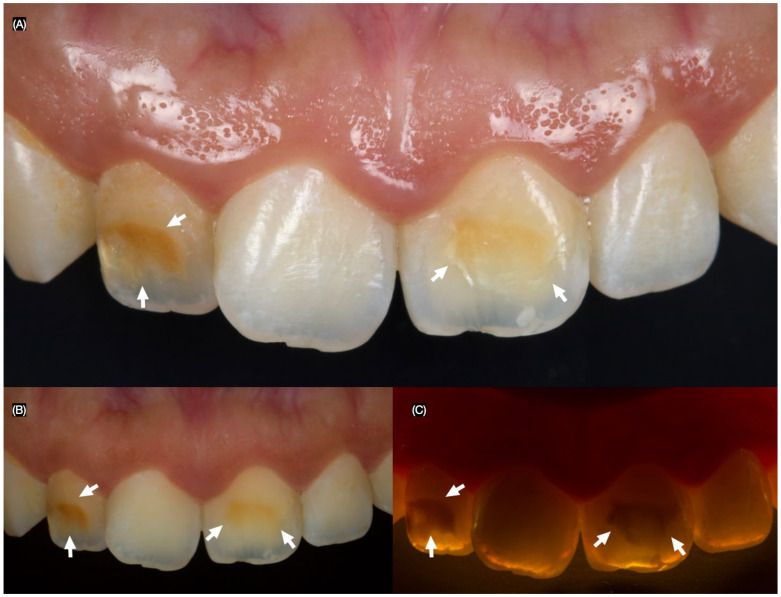
Initial intra-oral situation and the arrows indicate the stain areas. (**A**) Frontal, (**B**) frontal with polarized lens, and (**C**) frontal with flash in the back to assess the stains.

A 26-year-old female patient presented to the clinic with the chief complaint, “I do not like the dark stain on my front teeth.” After a clinical evaluation, the patient was diagnosed with a stained maxillary right lateral incisor, a stained maxillary left central incisor, non-ideal gingival architecture, and a tilted maxillary right central incisor (Figure 1).

The patient was offered orthodontic treatment to improve tooth alignment, along with crown lengthening to enhance the gingival architecture of the anterior teeth. This would be followed by tooth whitening and macroabrasion, along with resin infiltration and composite restorations on the right lateral incisor and left central incisor. However, the patient declined orthodontic treatment due to financial concerns, crown lengthening because of her dislike for surgical procedures, and tooth whitening to avoid potential tooth sensitivity. The patient only requested microabrasion with resin infiltration and resin composite restorations.

**Figure 2:**
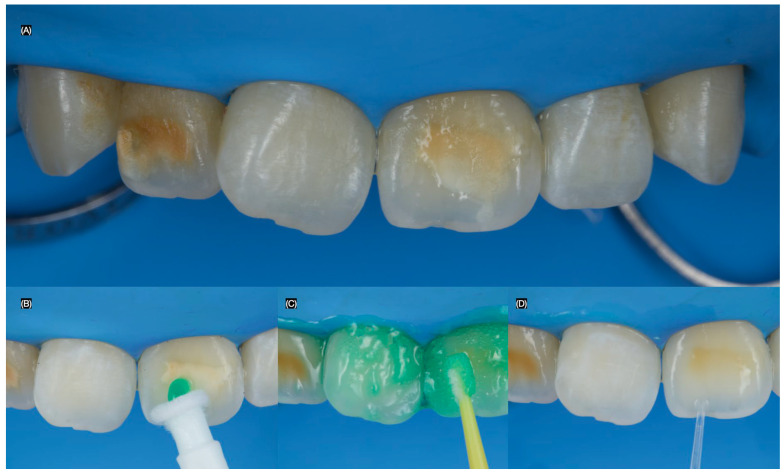
Resin infiltration procedure. (**A**) Initial dental dam isolation, (**B**,**C**) hydrochloric acid application, and (**D**) application of the infiltrating resin.

A dental dam (Dental Dam, Nic Tone, Bucharest, Romania) was placed from the maxillary left first premolar to the maxillary right first premolar and secured with clamps (Clamp #00, Hu-Friedy, Chicago, IL, USA) to ensure proper isolation. A 15% hydrochloric acid solution (Icon-Etch, DMG, Ridgefield Park, NJ, USA) was then applied to the teeth from the right to the left canine for 2 min, removed with high suction, and rinsed with water for 30 s. This procedure was repeated. Next, the infiltrating resin (Icon-Infiltrant, DMG, Ridgefield Park, NJ, USA) was applied to the surface and allowed to penetrate for 3 min, during which the surface was massaged with the applicator. The excess resin was removed with cotton pellets, and the tooth was light-cured for 40 s (Figure 2).

**Figure 3:**
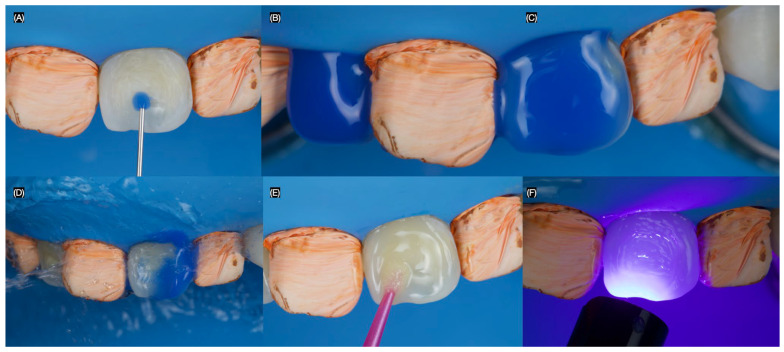
Macroabrasion. (**A**) Initial application of phosphoric acid for central incisor, (**B**) right lateral, and (**C**) left central incisors with phosphoric acid, (**D**) rinsing the acid, (**E**) adhesive application, and (**F**) light-curing.

A spherical diamond bur (Jota AG, Rüthi, Switzerland) was used to remove the stain on the maxillary right lateral incisor and left central incisor, with high irrigation to keep the teeth hydrated, without creating a defined tooth preparation outline. The right canine and left lateral incisors were isolated with Teflon tape. Then, 35% phosphoric acid (Ultra Etch, Ultradent, South Jordan, UT, USA) was applied to the right lateral incisor and left central incisor for 20 s, rinsed off, and air-dried. Next, adhesive (Optibond FL, Kerr Dental, Brea, CA, USA) was applied, excess was removed with air, and the surface was light-cured for 20 s (Figure 3).

**Figure 4:**
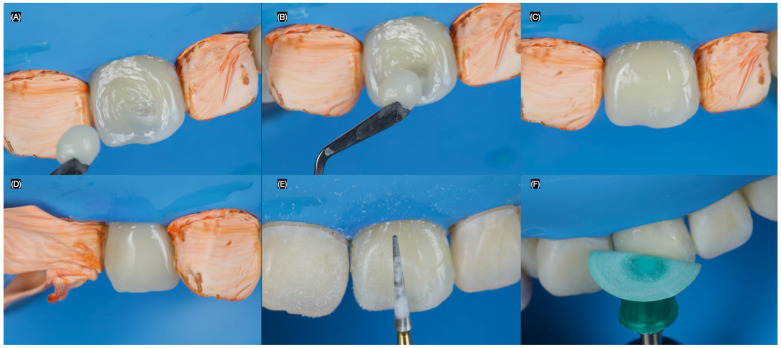
Restoration procedure. (**A**) Final microabrasion, (**B**) initial placement of the resin composite final application for the (**C**) central and (**D**) lateral incisors, (**E**) finishing, and (**F**) polishing of the restorations.

The stratification of the resin composite (Clearfil AP-X, Kuraray Dental, Tokyo, Japan) began with a thin layer of A2, followed by a layer of A1. The increments were polymerized using a curing light (Valo, Ultradent Products Inc., South Jordan, UT, USA) for 20 s. The restorations were finished with diamond burs (Composite Finishing Kit, Jota AG, Rüthi, Switzerland) and then polished using Professional Polishing Composite (Jota AG, Rüthi, Switzerland) (Figure 4).

**Figure 5:**
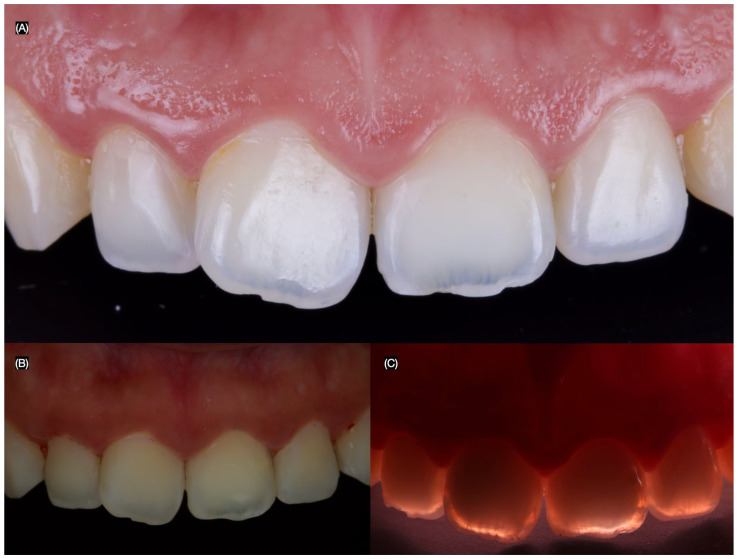
Final intra-oral situation. (**A**) Frontal view, (**B**) frontal view with polarized lens, and (**C**) frontal view with flashes in the back to assess the stain.

**Figure 6:**
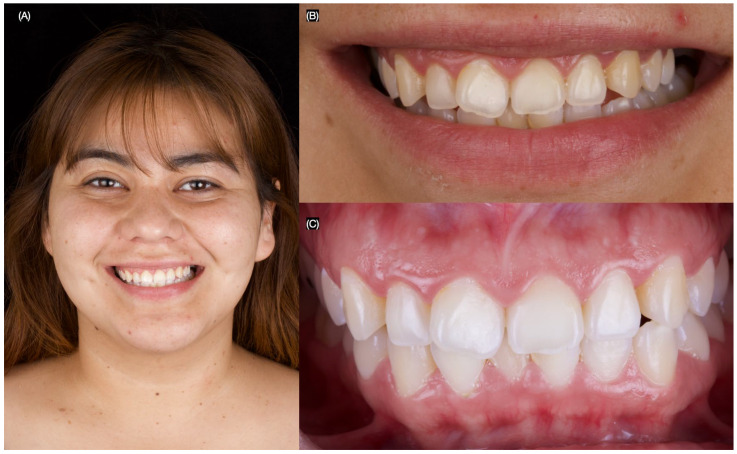
Final situation. (**A**) Patient smiling, (**B**) smile, and (**C**) teeth in occlusion.

The patient was pleased with the final contours, shape, and shade of restorations provided (Figure 5 and Figure 6).

The patient was provided with comprehensive oral hygiene instructions, including brushing three times daily and using dental floss regularly. She was scheduled for dental prophylaxis twice a year and was informed that the microabrasion treatment would be evaluated at each of those visits. The patient was also advised to improve her dietary habits by reducing sugar intake and avoiding foods and beverages that could stain the teeth. Additionally, she was informed that re-polishing of the restorations might be necessary if they became rough over time.

**Figure 7:**
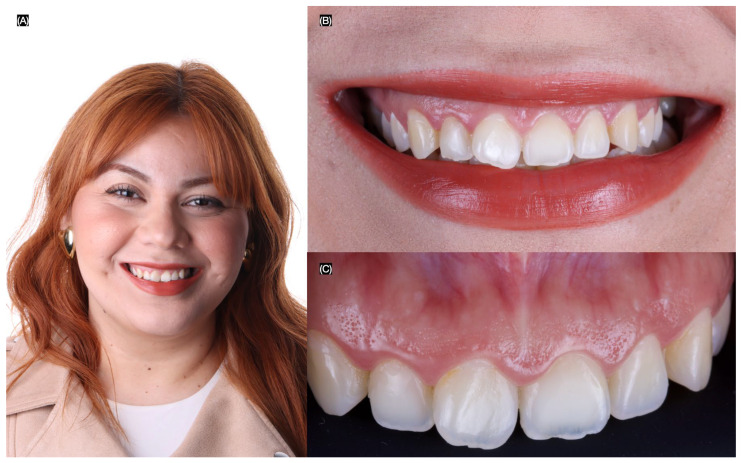
Three years follow-up. (**A**) Face smiling, (**B**) smile, and (**C**) intra-oral situation.

The patient fully understood the instructions and complied with the follow-up schedule, attending her appointments every six months. Throughout the follow-up period, re-polishing of the restorations was not required. At the three-year follow-up appointment, the patient remained satisfied with the esthetic outcome. Both the restorations and the periodontal tissues were found to be in clinically acceptable condition (Figure 7).

## 3. Results

### 3.1. Results of the Narrative Review

The literature includes some case reports on microabrasion and macroabrasion treatments; however, very few systematically describe each clinical step of the procedure. The findings from our brief narrative review of articles outlining the clinical steps for microabrasion and macroabrasion are based on seven publications, which are presented in Table 3 [11,21,22,23,24,25,26].

The findings from the literature review of retrospective clinical studies, including narrative reviews, scoping reviews, and systematic reviews evaluating the effectiveness of microabrasion and macroabrasion, are based on five studies, which are presented in Table 4 [13,27,28,29,30].

### 3.2. Results of the Case Study

The clinical workflow outlined in this case study began with careful planning and precise execution, with each step guided by the desired esthetic outcome. The treatment successfully met the patient’s expectations using a conservative approach. The macroabrasion technique used did not follow the extensive outlines seen in traditional preparations for crowns, veneers, or Class III/IV composite restorations, thereby preserving more natural tooth structure. While resin infiltration helped reduce the initial stain, it was not sufficient to achieve the desired result. Consequently, microabrasion using a diamond bur was performed. The placement of a resin composite in the ideal shade restored a natural-looking color, while finishing and polishing provided a smooth, lifelike contour. Additionally, the use of total isolation with a dental dam prevented contamination of the working field and enhanced the bonding performance of the restorative materials.

## 4. Discussion

### 4.1. Microabrasion

Microabrasion is a conservative, chemo-mechanical technique used in dentistry to remove superficial enamel discolorations and defects. This procedure involves the application of an acidic–abrasive compound—commonly a combination of hydrochloric acid and pumice or silicon carbide particles—applied directly to the enamel surface [28]. The material is typically delivered using a rubber cup attached to a low-speed handpiece, allowing controlled removal of the outermost enamel layer. The primary goal of microabrasion is to eliminate intrinsic or extrinsic stains limited to the superficial enamel without compromising the structural integrity of the tooth. Studies have demonstrated that microabrasion can safely remove between 25 and 200 µm of enamel, a range considered clinically acceptable and unlikely to significantly weaken the tooth [10].

Despite its conservative nature and esthetic benefits, microabrasion has certain limitations, particularly when dealing with deeper enamel discolorations or stains that extend into the dentin. For this reason, clinicians often combine microabrasion with adjunctive treatments such as in-office or at-home bleaching, and resin infiltration. This multimodal approach can enhance the esthetic outcome, especially in cases of fluorosis, white spot lesions, or developmental enamel defects. Moreover, microabrasion has been shown to improve surface smoothness and resistance to plaque accumulation, potentially aiding in long-term maintenance of oral hygiene and esthetics. Nonetheless, case selection and proper diagnosis are essential to ensure the procedure’s effectiveness and to set realistic expectations for patients.

### 4.2. Resin Infiltration

Resin infiltration has emerged as a minimally invasive and esthetically effective technique for the treatment of early enamel lesions, particularly in the management of white spot lesions [31]. These lesions, often resulting from enamel demineralization due to caries or orthodontic treatment, present a challenge for both clinicians and patients because of their visibility and resistance to traditional whitening treatments [32]. The infiltration process utilizes a specially formulated light-cured resin that features low viscosity and high surface tension. These properties enable the resin to deeply penetrate the porous structure of the demineralized enamel. By infiltrating and filling the microporosities, the resin modifies how light interacts with the enamel, effectively reducing the optical contrast between the lesion and the surrounding healthy tooth structure. This phenomenon, known as “color masking”, occurs because the refractive index of the infiltrated resin closely matches that of natural enamel, thereby minimizing light scattering within the lesion.

Before resin infiltration, a mild etching step is typically performed to remove the superficial layer of enamel, which acts as a barrier to resin penetration. This step allows for deeper and more uniform infiltration of the material into the lesion body. An added benefit of this approach is that it not only improves esthetics but also seals the porous enamel surface, potentially halting the progression of early carious lesions and offering some protection against future demineralization. Despite its advantages, questions remain regarding the long-term durability of resin infiltration. Some studies have raised concerns about its resistance to staining over time, its ability to maintain esthetic results, and the need for retreatment in certain cases [33]. Continued research is needed to evaluate its longevity, especially in patients with ongoing risk factors for enamel demineralization. Nonetheless, resin infiltration is widely regarded as a valuable option in the minimally invasive management of non-cavitated enamel lesions, offering both preventive and cosmetic benefits.

### 4.3. Macroabrasion

The macroabrasion procedure is a technique used to remove localized stains or white spot lesions. It is typically performed using finishing burs at high speed and low speed until the affected area is removed. Light, intermittent pressure is recommended to remove the dental structure without creating a specific cavity preparation shape [12]. Clinicians must ensure that the tooth remains hydrated during the procedure, as some white spot lesions may be susceptible to dehydration. Dehydrated lesions tend to exaggerate the appearance of white spots, making it more difficult to remove the defects [25]. A round, spherical, or elliptical diamond bur is recommended for preparing the tooth until the stain is removed. The preparation should be performed with air and water cooling to avoid overcontouring and to prevent involvement of subgingival or incisal angles [34]. Macroabrasion can be considered a fast, safe, and efficient alternative to enamel microabrasion and is commonly associated with tooth whitening and esthetic procedures [35,36].

### 4.4. Tooth Isolation

Tooth isolation plays a critical role in enhancing the success of dental procedures, offering a range of clinical advantages. One of its primary benefits is the prevention of contamination from saliva, blood, and gingival crevicular fluid, which can compromise bonding and other restorative procedures. Effective isolation also significantly improves visibility and access to the treatment area, allowing clinicians to perform with greater precision and efficiency [37]. The use of dental dams, in particular, has been well-supported in the literature. A 2006 systematic review published by the Cochrane Library found that isolating teeth with a rubber dam significantly reduces the failure rates of direct dental restorations, such as resin composites, compared to procedures performed without isolation [38]. This is attributed to the creation of a clean, dry working environment, which is essential for optimal adhesion and material performance.

Beyond clinical benefits, the use of dental dams has been associated with improved patient experience. Studies have shown that many patients report feeling more comfortable during treatments when a dental dam is used. The dam helps prevent debris, water spray, and dental materials from entering the mouth, which can reduce anxiety and enhance tolerance for longer procedures [39]. However, clinicians must be cautious when selecting the material for the dental dam. Traditional rubber dams made from latex can cause allergic reactions in sensitive individuals. Symptoms may range from mild irritation and skin redness to more severe reactions such as rashes, hives, itching in the nose, throat, or eyes, and even nausea. To prevent these complications, the use of non-latex (often nitrile-based) dental dams is strongly recommended, especially when a latex allergy is suspected or confirmed [40]. Overall, the routine use of dental dams in restorative and esthetic dentistry supports better clinical outcomes, enhances patient safety and comfort, and aligns with evidence-based best practices in modern dental care.

### 4.5. Case Report

This report aims to demonstrate that conservative treatment options, such as microabrasion, can be effective for patients presenting with stains on the anterior dentition. These techniques should be considered before traditional restorations like ceramic veneers or crowns, which require more defined and invasive tooth preparation.

In this case, the patient presented with esthetic concerns. Due to her young age, she was offered a conservative treatment plan involving resin infiltration followed by microabrasion. The patient was fully informed about the procedure, including its limitations. She was advised that there was no guarantee of complete stain removal, but the conservative approach could be attempted prior to more invasive treatments like veneers. She agreed to proceed. Patient education played a crucial role in managing expectations and avoiding potential disappointment if the stains could not be fully removed or masked with resin composite.

Following a review of the patient’s medical and dental history, no systemic condition was found to explain the anterior staining. This led clinicians to diagnose the stains as locally caused, likely due to dietary factors and poor oral hygiene. It was also determined that the stains had not penetrated deeply into the dentin. The initial resin infiltration significantly reduced both the size and intensity of the stains but did not eliminate them completely. Therefore, microabrasion was performed. Although this procedure involved the use of a high-speed diamond bur, it remained conservative, as only the stained areas were targeted without creating a defined preparation or outline. The procedure was performed under copious water cooling to prevent heat generation and protect the pulp from inflammation.

Accurate shade selection and placement of the resin composite were essential to achieving a natural-looking result. Finishing and polishing steps were equally important in reproducing the surface texture and gloss to match the adjacent teeth. All dental procedures were performed under total isolation with a dental dam. This ensured protection of the gingival tissues from acid exposure, safeguarded the patient’s airway from accidental ingestion of materials, and prevented contamination of the bonding surfaces. Proper isolation also optimized the bonding effectiveness of the restorative materials. After treatment, the patient was scheduled for recall visits every six months for dental prophylaxis and evaluation of the treatment outcomes. At the three-year follow-up, the patient remained satisfied with the esthetic results.

### 4.6. Precautions

Clinicians must take several precautions before and during the performance of micro- and macroabrasion procedures. First, it is important to explain to the patient that the success of these treatments depends on the depth of the stain or lesion. In cases where conservative treatments are not effective, traditional options such as veneers or crowns may be necessary. An initial tooth shade evaluation is strongly encouraged and should be documented with photographs. This allows for easy comparison of shade improvements after treatment and helps patients visually assess the results. It also assists clinicians in accurately selecting the appropriate tooth shade, in case macroabrasion is required. Shade selection should be performed while the teeth are still hydrated, before the placement of the dental dam.

Before beginning the procedure, both the patient and clinician should wear eye protection, and the use of a dental dam is strongly recommended to prevent exposure to the acid used during treatment. After the procedure, patients should be reminded that maintaining good oral hygiene is essential to preserve the results. This includes brushing three times per day, using dental floss, and attending professional dental cleanings twice a year. Patients should also be informed that the restorations will be evaluated at least once a year, and occasional re-polishing may be necessary.

### 4.7. Clinical Significance

The clinical significance of this narrative review on microabrasion and macroabrasion in dentistry lies in its comprehensive evaluation of the current evidence-based literature, which supports the effectiveness of these techniques for the conservative management of enamel discoloration, particularly in the anterior dentition. Both microabrasion and macroabrasion have been shown to be minimally invasive procedures that effectively remove superficial stains and enamel defects while preserving healthy tooth structure. These approaches are particularly beneficial for patients seeking esthetic improvement without the need for more aggressive restorative interventions.

Additionally, microabrasion and macroabrasion are recognized as safe treatment modalities with minimal adverse effects, making them suitable for a wide range of patients, including younger individuals. They are often considered as first-line options for addressing esthetic concerns related to superficial enamel opacities, fluorosis, and post-orthodontic stains. The clinical case study presented further enhances the practical relevance of this review by outlining the step-by-step protocol of a macroabrasion procedure. This case provides valuable insights for clinicians by demonstrating the application of the technique in a real-world setting, supported by a three-year follow-up. The long-term results confirm the durability and satisfaction associated with the treatment, with the patient continuing to express fulfillment with the esthetic outcome.

### 4.8. Limitations

This narrative review has some limitations, such as relying on a few databases (PubMed, Google Scholar, Semantic Scholar, and ResearchGate), searching in only one language (English), and using a focused search strategy, which does not align with the Preferred Reporting Items for Systematic Reviews and Meta-Analyses (PRISMA) guidelines. The limitations of the single case report include the lack of quantitative analysis, with the qualitative analysis based solely on the patient’s self-reported satisfaction with the results at the 3-year follow-up. Additionally, the case study is limited to a 3-year follow-up, so future case studies should include longer follow-up periods. Lastly, future research should also compare different adhesive brands and resin composites used in macroabrasion and microabrasion treatments.

## 5. Conclusions

Microabrasion is effective for superficial enamel stains, while macroabrasion is better suited for deeper defects. Accurate diagnosis is key to choosing the right treatment and setting patient expectations. In the case study, resin infiltration combined with macroabrasion achieved satisfactory esthetic results, maintained over a 3-year follow-up.

## Figures and Tables

**Table 1:** Etiology for intrinsic tooth discoloration [8].

Enamel	Dentin
Local Causes	Local Causes
Caries	Caries
Idiopathic	Metallic Restorative Materials
Injuries	Necrotic Pulp Tissue
Internal Resorption	Endodontic Filling Materials
Systemic Causes	Systemic Causes
Drugs (e.g., Tetracyclines)	Drugs (e.g., Tetracyclines)
Amelogenesis Imperfecta	Dentinogenesis Imperfecta
Fluorosis	Congenital Porphyria
Idiopathic	Bilirubin
Systemic Illness During Tooth Formation	

**Table 2:** Differences between micro- and macroabrasion [9,10,11,12,13].

Microabrasion	Macroabrasion
Removes super stain in enamel	Removed deep stain in enamel
Chemical + mechanical removal	Mechanical removal
Uses rubber cup on slow-speed handpiece	Uses high-speed handpiece with rotatory instruments
Performed with acid + abrasive slurry (e.g., 6–19 HCI + pumice)	Performed with fine-grit diamond bur or finishing discs
Removal of 0.2 mm (200 µm) tooth structure	Removal of 0.3–0.5 mm (300–500 µm) tooth structure

**Table 3:** Publications on clinical treatment providing micro- or macroabrasion in the esthetic zone [11,21,22,23,24,25,26].

Authors and Year of Publication	Title	Clinical Treatment	Conclusions
Pavani CC et al. 2021 [21]	Case Reports of Enamel Microabrasion Associated with At-home Dental Bleaching After Orthodontic Bracket Removal	Two reports treated as follows: After orthodontics, the treatment included at-home bleaching (10% carbamide peroxide) 6–8 h each day; after 1 week, microabrasion was performed.	The association of at-home dental bleaching with enamel microabrasion was effective for obtaining satisfactory shade alteration associated with a smooth and regular enamel surface after orthodontic bracket debonding, highly contributing to improved dental esthetics.
Sundfeld RH et al., 2014 [11]	Microabrasion in tooth enamel discoloration defects: three cases with long-term follow-ups	Stains on the maxillary incisors were removed by the enamel microabrasion technique with an application of 18% hydrochloric acid and pumice. Stains on the mandibular teeth were removed with an application of the enamel microabrasion product PREMA Compound (Premier).	Correct application of the microabrasion technique, complemented or not by the bleaching or the use of composite resin, allowed for significant improvement in the appearance and color uniformity of the teeth, restoring the patient’s self-esteem.
Balan B et al., 2013 [22]	Microabrasion: an effective method for improvement of esthetics in dentistry	Two case reports treated first with 18% hydrofluoric, then cleaned with pumice paste and finally polished with porcelain cup-shaped polishing rubber.	For mild fluorosis discoloration and for moderate/severe fluorosis, treatment to change the esthetic appearance of the teeth can be accomplished with minimally invasive treatment using microabrasion, or in case of moderate–severe condition, combinations of microabrasion with bleaching can be performed to provide the patient with an esthetically acceptable result.
Pontes DG et al., 2012 [23]	Re-establishing esthetics of fluorosis-stained teeth using enamel microabrasion and dental bleaching techniques	An 18-year-old female patient with moderate fluorosis received enamel microabrasion with 6% hydrochloric acid followed by in-office bleaching.	Microabrasion and bleaching are painless, fast, and easy to perform, in addition to preserving the dental structure. Treatment showed immediate and permanent results; this technique must be promoted among professionals and their patients.
Nicholas LS et al., 2023 [24]	Conservative esthetic management of brown enamel fluorosis using combination therapy: A clinical report	A 19-year-old female patient with brown spots in the front teeth. Treatment included 15% hydrochloric acid, then cleaning with pumice paste, and then air microabrasion was provided, followed by tooth bleaching (35% carbamide peroxide) and finally resin infiltration (ICON).	The combination approach of enamel microabrasion, bleaching, and resin infiltration techniques can be used in the management of more severe types of intrinsic tooth discoloration.
Gaião U et al., 2022 [25]	Macroabrasion and/or Partial Veneers: Techniques for the Removal of Localized White Spots	A 16-year-old female patients with hypoplastic spots on the upper central incisors. Treatment included macroabrasion and partial veneer technique direct composite resin.	The association of enamel macroabrasion and partial veneer in composite resin is conservative, fast, and an esthetic alternative for removing localized hypoplasia stains.
Jyothi M et al., 2016 [26]	Conservative Management of Discoloured Anterior Teeth—A Case Series	Three case reports of patients presenting brownish discoloration involving the anterior teeth. Treatment included microabrasion with hydrochloric acid (Opalustre), followed by microabrasion with finishing bur, and then teeth were cleaned with polishing paste and finally restored with fluoride varnish.	The improvements made by the conservative esthetic dentistry represents a new dimension of dental treatment for patients. The restoration of a smile is one of the most appreciated and gratifying services a dentist can render.

**Table 4:** Reviews evaluating the success of micro- or macroabrasion [13,27,28,29,30].

Authors and Year of Publication	Title	Findings	Conclusions
Pini NI et al., 2015 [13]	Enamel microabrasion: An overview of clinical and scientific considerations.	The most important factors contributing to the success of enamel microabrasion are the location and depth of the enamel stain or defect. The alteration must be restricted to enamel tissue, without involvement of the dentin. Deeper, opaque stains, such as those resulting from hypoplasia, cannot be resolved with microabrasion and require a restorative approach.	Accumulating evidence suggests that enamel microabrasion is efficient and effective for producing esthetic improvements. This technique involves minimal enamel loss, leaving a smooth and shiny enamel surface with permanent results.
Blanchet I et al., 2023 [27]	Microabrasion in the management of enamel discolorations in paediatric dentistry: a systematic review.	The main assessment used in the included studies was an improvement in smile esthetics after microabrasion treatment. The initial description of the discoloration follows a clinical examination allowing comparison with different classifications (TF Index, Dean Index, TSIF, ICDAS, MDDEI, DDE index, MIH EAPD classification) without precise depth assessment.	Microabrasion appears to be an effective and reliable technique for the management of pre- and post-eruptive enamel discolorations of permanent teeth in pediatric dentistry, especially for dental fluorosis for which this review shows an efficiency on the mild to severe forms.
Rocha AO et al., 2024 [28]	A global overview of enamel microabrasion for white spot lesions: a bibliometric review.	Regarding the microabrasion protocol, Opalustre (Ultradent) was the most used material, followed by comparisons between different materials, pumice stone and 37% phosphoric acid, Prema (Premiere), hydrochloric acid and pumice stone, hydrochloric acid alone, acid and sandpaper disk, diamond tip and acid, and laser.	The scientific background of enamel microabrasion for the treatment of white spots is based mainly on case reports and series. The most addressed white spot lesion in the studies was dental fluorosis, and the main objective of the studies was to evaluate the clinical performance of this procedure.
Bacionyte G et al., 2019 [29]	Microabrasion in Pediatric Dentistry and Orthodontics	Microabrasion can only remove lesions located not deeper than in the 40 um depth of enamel, so this kind of treatment for deeper lesions is not effective enough and so have to be combined with other treatment methodologies. Precise lesion depth evaluation in vivo can be only evaluated using traditional methodologies, e.g., ultrasound, transillumination or visual evaluation, which is not always precise enough.	Every clinical situation requires analytical thinking and complex treatment plan. It was already proven that any kind of treatment for patients with enamel defects can be useful to increase the quality of the social life so it is always good to consider microabrasion as a minimally invasive treatment to benefit for the following treatment of enamel defects.
Di Giovanni T et al., 2018 [30]	Interventions for dental fluorosis: A systematic review	Compared to bleaching, microabrasion resulted in a significantly smaller esthetic improvement of fluorotic stains 6 months after treatment, which was clinically relevant. On the other side, no difference could be found post-treatment in tooth sensitivity between the microabrasion and bleaching.	Based on the existing limited evidence, resin infiltration seems to be the most promising treatment for dental fluorosis, followed by bleaching and microabrasion.

## Data Availability

Data presented in this study are available on request from the corresponding author.
